# The Challenging Heterogeneity of Autism: Editorial for Brain Sciences Special Issue “Advances in Autism Research”

**DOI:** 10.3390/brainsci10120948

**Published:** 2020-12-07

**Authors:** Antonio Narzisi

**Affiliations:** Department of Child Psychiatry and Psychopharmacology, IRCCS Stella Maris Foundation, 56018 Pisa, Italy; antonio.narzisi@fsm.unipi.it

## 1. Introduction

My personal experience as Guest Editor of the Special Issue (SI) entitled “Advances in Autism Research” began with a nice correspondence with Andrew Meltzoff, from the University of Washington, Seattle (WA, USA), which, in hindsight, I consider as a good omen for the success of this Special Issue: “*Dear Antonio….I am happy you are editing a special issue on this important topic. It will be useful for society, as well as for psychology, neuroscience, and of course the children and families…….Best, Andy.”*

Advances in Autism Research was a unique experience from a scientific and human point of view; 50 contributions took part in this SI from five Continents (see Appendix A). Globally, all the articles involved 356 authors and 156 reviewers between researchers and clinicians actively engaged in the field of autism spectrum disorder (ASD). 

The SI saw, alongside a significant representation of the major Italian institutions that deal with ASD, the contribution of worldwide recognized experts in ASD such as Professor Sally Rogers from MIND Institute, CA, USA.

The SI took place during a very important historical period for the world community; the COVID-19 pandemic has profoundly changed our way of living every day as human beings and the way in which we are carrying out our work as researchers and clinicians. 

The SI addressed many topics including: (1) COVID-19 pandemic; (2) Epidemiology and prevalence; (3) Screening and early behavioural markers; (4) Diagnostic and phenotypic profile; (5) Treatment and intervention; (6) Etiopathogenesis (Biomarkers, Biology, Genetic, Epigenetic and Risk Factor); (7) Autism and comorbidity; (8) Autism and adulthood; and (9) Broader Autism Phenotype (BAP).

## 2. COVID-19 Pandemic

The SI hosted three papers that described the COVID pandemic. First, Narzisi’s editorial [1] whose goal was to help clinicians and parents manage the difficult moment of the lockdown that people with autism and their families have had to endure. Second, the research paper of Colizzi and colleagues [2] aimed to investigate the impact of the COVID-19 pandemic on ASD individuals. COVID-19 emergency resulted in a challenging period for 93.9% of families, increased difficulties in managing daily activities, especially free time and structured activities and children presenting with more intense and more frequent behavior problems. Third, the retrospective study of Brondino and colleagues [3] evaluated the impact of COVID-19 restrictions on challenging behaviors in a cohort of people with severe ASD attending a daycare center at the beginning of the pandemic. Authors showed that during the first two weeks of the pandemic, there were no observed variations in challenging behaviors. This suggested that adaptations used to support individuals with ASD in adapting to the COVID-19 emergency restrictions were effective for managing their behavior.

## 3. Epidemiology and Prevalence

In the field of epidemiology, Chiarotti and Venerosi [4], from the Italian National Institute of Health, presented an interesting review of the ASD prevalence estimates published since 2014. Data confirmed a high variability in prevalence across the world, likely due to methodological differences in case detection, and the consistent increase of prevalence estimates within each geographical area. 

## 4. Screening and Early Behavioural Markers

The SI included six papers about the screening of ASD. Petrocchi and colleagues’ study [5] provided a systematic review of level 1 and level 2 screening tools for the early detection of ASD under 24 months of age. Levante and colleagues [6] examined the cross-cultural generalisability of the First Year Inventory (FYI) on an Italian sample, testing its construct validity, consistency, and structural validity. Their findings supported the generalisability of the Italian version of the FYI and its validity. Devescovi and colleagues [7] aimed to identify early signs of atypical development consistent with ASD or other developmental disorders in a population of 224 low-risk toddlers through a two-stage screening approach applied at 12 and 18 months of age using the Communication and Symbolic Behavior Scales Developmental Profile (CSBS DP) Infant–Toddler Checklist (I-TC) and the Quantitative Checklist for Autism in Toddlers (Q-CHAT). Their results showed that autistic signs can be detected as early as the first year even though a few questions extrapolated from both screeners with potential benefit in terms of the screening procedure. Taresh and his group [8] presented a conceptual framework aimed to utilize the current literature to present a discussion on preschool teachers’ knowledge, belief, identification skills, and self-efficacy (KBISSE) in identifying children with ASD and making decisions to refer children suspected with ASD to specialists. The conceptual framework emphasized the need for preschool teachers to be educated in ASD via an educational module that could increase teachers’ self-efficacy in identifying children with ASD. Finally, the studies of Baccinelli [9] and Caruso [10], coordinated by Maria Luisa Scattoni from the Italian National Institute of Health, were aimed to investigate the early motor trajectories of infants at high-risk (HR) of ASD through MOVIDEA, a semi-automatic software developed to analyze 2D and 3D videos and provide objective kinematic features of their movements. Results revealed that early developmental trajectories of specific motor parameters were different in HR infants later diagnosed with Neuro-Developmental Disorders from those of infants developing typically.

## 5. Diagnostic and Phenotypic Profile

### 5.1. Motor Profile

Alsaedi’s study [11] aimed to determine the prevalence, severity, and nature of the motor abnormalities in children with ASD as well as to elucidate the associated developmental profiles. The short-form of the Bruininks–Oseretsky Test of Motor Proficiency, Second Edition (BOT-2) was used to assess various aspects of the motor performance of children with ASD and typically developing from three Gulf states. The results revealed the high prevalence of motor abnormalities among the ASD group when compared with the normative data derived from the BOT-2 manual as well as with the data concerning the typically developing group.

### 5.2. Language Profile

Barsotti and colleagues [12] aimed to study the grammatical comprehension of children with ASD. The presence of receptive difficulties in school-age ASD children with relatively preserved non-verbal cognitive abilities could provide important hints to establish rehabilitative treatments. The main finding of the study showed that language comprehension is the most impaired language domain in ASD. The Gladfelter and Barron’s study [13] explored whether global–local processing differences influence the type of semantic features children with ASD, developmental language disorder (DLD), and their neurotypical peers learn to produce when learning new words. Results indicated that the children with ASD and DLD produced more global, rather than local, semantic features in their definitions than the children with typical language. Shield [14] presented a longitudinal case study of a single child with ASD, a hearing, signing child of Deaf parents. Lexical signs and fingerspelled letters were coded for the four parameters of sign articulation (handshape, location, movement, and palm orientation). Longitudinal data suggested that palm orientation errors could be rooted in both imitation differences and motoric difficulties.

### 5.3. Neuropsychological Profile

Cristofani and colleagues [15] aimed to examine the role of empathic dimensions and executive skills in regulating externalizing behaviors. This study further corroborated developmental models of empathy and their clinical implications, for which externalizing behaviors could be attenuated by enhancing executive functioning skills.

The aim of the Andreou and Skrimpa’s review [16] was to present the most recent available studies with respect to the connection between the function of mirror neurons in individuals with ASD and ToM-reflecting sensorimotor, social and attentional stimuli. The majority of these studies approach the theory of broken mirror neurons critically. Findings from electroencephalography (EEG) studies so far indicate that further research is necessary to shed more light on the mechanisms underlying the connection(s) between ToM and neurophysiological operations.

The study of Papangelo and the group of Maddalena Fabbri-Destro [17], from the Institute of Neurology of National Council of Research of Parma, was aimed to evaluate the performance of human figure drawings (HFD) of children with ASD relative to typically developing (TD) controls. Findings suggested that the use of HFD tests with individuals with ASD may not be used in clinical practices. However, in basic research, HFDs could be used to highlight dependencies between drawing performance and neuropsychological features, thus possibly providing hints on the functioning of autism.

### 5.4. Sensory Profile

The study of Panerai and colleagues [18] was aimed to better understand the relationship between sensory and feeding problems in ASD by comparing the sensory responsiveness of ASD children with (ASD-W) and without (ASD-WO) feeding problems. Both groups showed strengths in Visual/Auditory sensitivity, Low-Energy/Weak, and Movement sensitivity, again more marked in ASD-WO. The work of Perin and his group [19] was aimed to investigate the Physiological Profile Assessment (PPA) in children and adolescents with ASD compared with age-matched typically developing (TD) individuals and examine the relationship between the PPA subset within the ASD and TD participants according to different age groups. Performance in most of the PPA tests significantly improved with older age in the TD group but not in the ASD group. Molinaro and colleagues [20] conducted a review of visual impairments in ASD. They highlighted the finding that in the absence of a valid methodology adapted for the visually impaired population, diagnosis of ASD in children with VI is often based on non-objective clinical impression, with inconclusive prevalence data. The study of Valori and colleagues [21] assessed the feasibility and offered some early insights from a new paradigm for exploring how children and adults with ASD interact with Reality and Immersive Virtual Realities (IVR) when vision and proprioception are manipulated. The pilot indicates the good feasibility of the paradigm. Preliminary data visualisation suggests the importance of considering inter-individual variability.

### 5.5. Migrant Background

The study of Garcia-Primo and colleagues [22] explored (i) differences in age at ASD diagnosis between children with and without a migrant background in the main diagnostic centre for ASD in Upper Austria (ii) factors related to the age at diagnosis and (iii) whether specific factors differed between the two groups. No delay in diagnosing ASD in children with a migrant background in a country with universal health care and an established system of paediatric developmental surveillance was found. Awareness of ASD, including Asperger’s syndrome, should be raised among families and healthcare professionals.

## 6. Treatment and Intervention

Fuller and the group of MIND Institute directed by Sally Rogers [23] conducted a meta-analysis in order to examine the effects of the Early Start Denver Model (ESDM) for young children with ASD on developmental outcome measures. Findings showed improvements in cognition and language. No significant effects were observed for measures of autism symptomology, adaptive behavior, social communication, or restrictive and repetitive behaviors. Marino and colleagues [24] presented an interesting RCT on telehealth. It was aimed at comparing the effect of a tele-assisted and in-person intervention based on a behavioral intervention protocol for families with children affected by ASDs. 

Substantial improvements in the perception and management of children’s behavior by parents, as well as in the influence of a reduction in parent stress levels on said children’s behavior through the use of a tele-assisted intervention, were obtained. This trial demonstrated the evidence-based potential for telehealth to improve the treatment of ASDs. Bentenuto and colleagues [25] investigated intervention effects in terms of mediators and moderators in order to explain the variability and to highlight mechanisms of change in children with ASD. The findings support the importance of parental involvement in targeting ASD core symptoms. Further, results informed our understanding of early predictors in order to identify specific elements to be targeted in the individualized intervention design. Yazdani and colleagues [26] conducted an important review to evaluate the early behavioral intervention studies of ASD based on their participant exclusion criteria. Results indicated that studies that used restrictive exclusion criteria demonstrated greater differences in terms of outcomes between experimental and control groups in comparison to studies that used loosely defined exclusion criteria and/or did not define any exclusion criteria. The authors described implications for the generalizability of the studies’ outcomes in relation to exclusion criteria. The study of Baker and colleagues [27] measured the reward positivity (RewP) in response to social and nonsocial stimuli in seven adolescents with ASD before and after participation in the Program for the Education and Enrichment of Relational Skills (PEERS®) intervention. Findings have implications for how neuroscience can be used as an objective outcome measure before and after intervention in ASD. Melongo and colleagues [28] reported a single case in which an intervention implemented to assist a 13.2-year-old boy with ASD without intellectual disability, aimed at improving his ability to compose persuasive texts was described. The Billeci and colleagues’ paper [29] was aimed to evaluate the process applied in subjects with ASD to elaborate and communicate their experiences of daily life activities, as well as to assess the autonomic nervous system response that subtends such a process. This was a proof-of-concept study on the application of the cognitive–motivational–individualized (c.m.i.®), which needs to be extensively validated in the clinical setting. In terms of general care treatment, Narzisi and colleagues [30] described an experience of dental care supported by Information and Communication Technologies (ICT), for children with ASD in a public health service. The project demonstrated acceptability by parents, suggesting that public health dental care and prevention can be successfully implemented without resorting to costly pharmacological interventions (with potential side effects), taking better care of children’s health.

## 7. Etiopathogenesis: Biomarkers, Biology, Genetic, Epigenetic and Risk Factor

Troisi and his group [31] described GEMMA (Genome, Environment, Microbiome and Metabolome in Autism), a prospective study supported by the European Commission, that follows at-risk infants from birth to identify potential biomarker predictors of ASD development followed by validation on large multi-omics datasets. The project includes clinical and pre-clinical studies in humanized murine models and in vitro colon models. This study will support the progress of a microbiome-wide association study (of human participants) to identify prognostic microbiome signatures and metabolic pathways underlying mechanisms for ASD progression and severity and potential treatment response.

Magdalena and colleagues [32] studied the preconception risk factors that are still poorly understood. The authors considered thirteen parameters for conception problems, conception with assisted reproductive techniques, the use and duration of oral contraception, the number of previous pregnancies and miscarriages, time since the previous pregnancy (in months), the history of mental illness in the family (including ASD), other chronic diseases in the mother or father and maternal and paternal treatment in specialist outpatient clinics. Findings showed that three factors statistically significantly increased the risk of developing ASD: mental illness in the mother/mother’s family, maternal thyroid disease and maternal oral contraception. Pascucci and colleagues [33], coordinated by Antonio M. Persico from University Hospital of Messina (Italy), assessed the effects of a single acute injection of low- or high-dose of p-cresol in behavioral and neurochemical phenotypes of BTBR mice, a reliable animal model of human ASD. Findings support a gene–environment interaction model, whereby p-cresol, acting upon a susceptible genetic background, can acutely induce autism-like behaviors and produce abnormal dopamine metabolism in the reward circuitry. Lombardo and colleagues [34] aimed to evaluate markers of infections and immune activation in ASD by performing a meta-analysis of publicly available whole-genome transcriptomic datasets of brain samples from autistic patients and otherwise normal people. Overall, the data did not support an association between infection and ASD. Prosperi and his group [35] investigated the role of inflammatory biomarkers in ASD and their correlations with clinical phenotypes. The results did not highlight the presence of any systemic inflammatory state in ASD subjects neither disentangling children with/without GI symptoms. Lee and colleagues [36] aimed to verify noteworthy findings between genetic risk factors and ASD by employing the false-positive report probability (FPRP) and the Bayesian false-discovery probability (BFDP). In this study, the authors found noteworthy genetic comparisons highly related to an increased risk of ASD. Multiple genetic comparisons were shown to be associated with ASD risk. The Caria and colleagues’ review [37] aimed to provide a critical synthesis of evidence linking alterations of the hypothalamus with impaired social cognition and behavior in ASD by integrating results of both anatomical and functional studies in individuals with ASD as well as in healthy carriers of oxytocin receptor (OXTR) genetic risk variant for ASD. Findings indicated that morphofunctional anomalies are implicated in the pathophysiology of ASD and call for further investigations aiming to elucidate anatomical and functional properties of hypothalamic nuclei underlying atypical socioemotional behavior in ASD. The review of Fusar-Poli and colleagues [38] was aimed to summarize the literature regarding the use of cannabinoids in ASD. The findings were promising, as cannabinoids appeared to improve some ASD-associated symptoms, such as problem behaviors, sleep problems, and hyperactivity, with limited cardiac and metabolic side effects. Conversely, the knowledge of their effects on ASD core symptoms is scarce. Tanaka and colleagues [39] in their pilot study focused on the neuroendocrinological response to participatory art activities, which are known to have a positive effect on emotion, self-expression, sociability, and physical wellbeing. These preliminary results suggested that the beneficial effects of participatory art activities may be partially mediated by oxytocin release, and may have therapeutic potential for disorders involving social dysfunction. The Stella Maris group [40] examined toddlers at their first diagnosis and after six months during two initiating joint attention (IJA) tasks using eye tracking. Findings suggest the potential use of eye-tracking technology as an objective, biological oriented marker, non-intrusive, adjunctive tool to measure developmental trajectories in toddlers with ASD.

## 8. Autism and Comorbidity

Masi and colleagues [41] conducted an exploratory study that addressed increased risk for suicidal ideation in high functioning autism spectrum disorders (HF-ASD). They studied this issue in a clinical group of 70 inpatient adolescents referred to a psychiatric emergency unit. Adolescents with Bipolar Disorder (BD) and HF-ASD and severe suicidal ideation or attempts (BD-ASD-S), were compared to adolescents with BD and HF-ASD without suicidal ideation or attempts (BD-ASD-noS), and to adolescents with BD and suicidal ideation or attempts without ASD (BD-noASD-S). Individuals with BD-ASD-S had a higher intelligence quotient, more severe clinical impairment, more lethality in suicide attempts, more internalizing symptoms, less impulsiveness, and lower social competence. The severity of ASD traits in individuals and parents did not correlate with suicidal risk. Some dimensions of resilience were protective in terms of repulsion by life and attraction to death. Gulisano and colleagues [42] aimed to identify the incidence of ASD in a large clinical sample of individuals affected by Gilles de la Tourette syndrome (GTS). Findings showed that the incidence of GTS with ASD was significantly lower in children than in adolescents. The incidence of GTS and ASD comorbidity in this study was high, and this has several implications in terms of treatment and prognosis. 

## 9. Autism and Adulthood

Griffiths and colleagues [43] developed an online survey instrument to assess employers’ perspectives on hiring job candidates with ASD. The cluster analysis indicated that company structures, policies and practices, and perceptions, as well as the needs of employers and employees, were important in determining who would successfully hire individuals with ASD. Key areas that require focused policies and practices include recruitment and hiring, training, accessibility and accommodations, and retention and advancement. Damiani and colleagues [44] aimed to test the association between epilepsy and regressive ASD. Secondly, they explored differences in behavioral and pharmacological profiles related to the presence of each of these conditions, as worse behavioral profiles have been separately associated with both epilepsy and regressive ASD in previous studies. The preliminary results suggested the presence of specific associations of different clinical conditions in subjects with rarely investigated phenotypes. In their paper, Keller and colleagues [45] described the experience of the Regional Center for Autism in Adulthood in Turin, Italy. It sought to develop a personalized rehabilitation and enablement program for people with ASD who received a diagnosis of autism in childhood/adolescence or for individuals with suspected adulthood ASD. This program is based on a Multistep Network Model involving people with ASD, family members, social workers, teachers, and clinicians. Findings indicated that the development of public centers specialized in assisting and treating people with autism (PWA) can improve the accuracy of ASD diagnosis in adulthood and foster specific habilitative interventions aimed to improve the quality of life of both PWA and their families. The study of Runge and colleagues [46] retrospectively analyzed the Cerebrospinal fluid (CSF) findings of adult patients with ASD. CSF basic measures (white blood cell count, total protein, albumin quotient, immunoglobulin G (IgG) index, and oligoclonal bands) and various antineuronal antibodies were compared with an earlier described mentally healthy control group of patients with idiopathic intracranial hypertension. The results of the study were limited by its retrospective and open design. The group differences in blood–brain barrier markers could be influenced by a different gender distribution between ASD patients and controls. The paper of Fusar-Poli and colleagues [47] was aimed to investigate self-reported autistic traits in individuals with ASD, schizophrenia spectrum disorders (SSD), and non-clinical controls (NCC), using the Autism-Spectrum Quotient (AQ) questionnaire. Findings showed that the AQ did not correlate with clinician-rated ADOS-2 scores in the ASD sample. Results confirmed that symptoms are partially overlapping in adults with ASD and psychosis. Moreover, they raise concerns regarding the usefulness of AQ as a screening tool in clinical populations

## 10. Broader Autism Phenotype (BAP)

Leonardi and colleagues [48] explored the construct of alexithymia in parents of children with and without ASD using a multi-method approach based on self-rated and external rater assessment. Results suggested the importance of using multi-method approaches to control for potential measurement bias and to detect psychological constructs such as alexithymia in subclinical samples such as parents of children with ASD. Riva and colleagues [49] conducted a prospective study of typically developing infants and measured frontal asymmetry in alpha oscillation (FAA) as a mediator between both maternal and paternal autistic traits and child ASD traits. Findings showed a potential cascade of effects whereby paternal autistic traits drive EEG markers contributing to ASD risk. Bianco and the group coordinated by Cosimo Urgesi [50] investigated whether the distribution of autistic traits in the general population, as measured through the Autistic Quotient (AQ), is associated with alterations of context-based predictions of social and non-social stimuli. Findings showed that the prediction of both social and non-social stimuli was facilitated when embedded in high-probability contexts. However, only the contextual modulation of non-social predictions was reduced in individuals with lower “Attention switching” abilities. The results provide evidence for an association between weaker context-based expectations of non-social events and higher autistic traits.

## 11. Conclusions

The published papers in this Special Issue (SI) testify to the complexity of performing research in the field of ASD. The multifactorial etiology inevitably calls different professional figures to a close collaboration. The published contributions underlined areas of progress and ongoing challenges which in the coming years could be able to give us more certain data.

To conclude, a special thank you to all authors who submitted their work to this Special Issue “Advances in Autism Research” and also the reviewers for dedicating their time and for helping to improve the quality of the published manuscripts.

Taking up the words of Andrew Meltzoff, who opened this editorial, the wish for this SI is that it might be, in its small way, *useful for society, as well as for psychology, neuroscience, and of course the children and families.*

## Appendix A

**Table A1:** Authors, Countries, Intitutions and Type of the papers included in the Special Issue “Advances in Autism Research”.

Papers Reference	Authors	Country	Institutions	Type
[1]	Narzisi A.	Italy	IRCCS Stella Maris	Editorial
[2]	Colizzi, M.; Sironi, E.; Antonini, F.; Ciceri, M.L.; Bovo, C.; Zoccante, L.	Italy	University of Verona	Research
[3]	Brondino, N.; Damiani, S.; Politi, P.	Italy	University of Pavia	Research
[4]	Chiarotti, F.; Venerosi, A.	Italy	Italian National Institute of Health	Review
[5]	Petrocchi, S.; Levante, A.; Lecciso, F.	Switzerland; Italy	University of Salento/Università della Svizzera Italiana	Review
[6]	Levante, A.; Petrocchi, S.; Massagli, A.; Filograna, M.R.; De Giorgi, S.; Lecciso, F.	Italy; Switzerland	University of Salento/Università della Svizzera Italiana/IRCCS European Institute of Oncology	Research
[7]	Devescovi, R.; Monasta, L.; Bin, M.; Bresciani, G.; Mancini, A.; Carrozzi, M.; Colombi, C.	Italy; USA	IRCCS Burlo Garofalo/University of Michigan/IRCCS Stella Maris	Research
[8]	Taresh, S.; Ahmad, N.A.; Roslan, S.; Ma’rof, A.M.; Zaid, S.	Malaysia; Yemen	University Putra Malaysia/Sana’a University	Review
[9]	Baccinelli, W.; Bulgheroni, M.; Simonetti, V.; Fulceri, F.; Caruso, A.; Gila, L.; Scattoni, M.L.	Italy	Italian National Institute of Health	Research
[10]	Caruso, A.; Gila, L.; Fulceri, F.; Salvitti, T.; Micai, M.; Baccinelli, W.; Bulgheroni, M.; Scattoni, M.L.; on behalf of the NIDA Network Group	Italy	Italian National Institute of Health	Research
[11]	Alsaedi, R.H.	Australia; Saudi Arabia	Queensland University of Technology/Taibah University	Research
[12]	Barsotti, J.; Mangani, G.; Nencioli, R.; Pfanner, L.; Tancredi, R.; Cosenza, A.; Sesso, G.; Narzisi, A.; Muratori, F.; Cipriani, P.; Chilosi, A.M.	Italy	IRCCS Stella Maris	Research
[13]	Gladfelter, A.; Barron, K.L.	USA	Northern Illinois University	Research
[14]	Shield, A.; Igel, M.; Randall, K.; Meier, R.P.	USA	Miami University/University of Texas	Research
[16]	Andreou, M.; Skrimpa, V.	Germany	University of Cologne	Review
[17]	Papangelo, P.; Pinzino, M.; Pelagatti, S.; Fabbri-Destro, M.; Narzisi, A.	Italy	National Research Council	Research
[18]	Panerai, S.; Ferri, R.; Catania, V.; Zingale, M.; Ruccella, D.; Gelardi, D.; Fasciana, D.; Elia, M.	Italy	IRCCS Research Oasi	Research
[19]	Perin, C.; Valagussa, G.; Mazzucchelli, M.; Gariboldi, V.; Cerri, C.G.; Meroni, R.; Grossi, E.; Cornaggia, C.M.; Menant, J.; Piscitelli, D.	Italy; Luxemburg; Canada; Australia; USA	University of Milano Bicocca/Villa Santa Maria Foundation/ASST Rhodense, Ospedale “G. SalviniP/LUNEX International University of Health, Exercise and Sports/University of New South Wales/McGill University/Pacific University	Research
[20]	Molinaro, A.; Micheletti, S.; Rossi, A.; Gitti, F.; Galli, J.; Merabet, L.B.; Fazzi, E.M.	Italy; USA	University of Brescia/ASST Spedali Civili of Brescia/Harvard Medical School	Review
[21]	Valori, I.; Bayramova, R.; McKenna-Plumley, P.E.; Farroni, T.	Italy; UK	University of Padova/Queen’s University Belfast	Research
[22]	Garcia Primo, P.; Weber, C.; Posada de la Paz, M.; Fellinger, J.; Dirmhirn, A.; Holzinger, D.	Austria; Spain	Johannes Kepler University/University of Education Upper Austria/Instituto de Salud Carlos III/University of Vienna/University of Graz	Research
[23]	Fuller, E.A.; Oliver, K.; Vejnoska, S.F.; Rogers, S.J.	USA	University of California, Davis MIND Institute	Review
[24]	Marino, F.; Chilà, P.; Failla, C.; Crimi, I.; Minutoli, R.; Puglisi, A.; Arnao, A.A.; Tartarisco, G.; Ruta, L.; Vagni, D.; Pioggia, G.	Italy	National Research Council	Research
[25]	Bentenuto, A.; Bertamini, G.; Perzolli, S.; Venuti, P.	Italy	ODFLAB, University of Trento	Research
[26]	Yazdani, S.; Capuano, A.; Ghaziuddin, M.; Colombi, C.	Usa	Loyola University/University of Michigan	Review
[27]	Baker, E.; Veytsman, E.; Martin, A.M.; Blacher, J.; Stavropoulos, K.K.M.	Usa	University of California	Brief report
[28]	Melogno, S.; Pinto, M.A.; Ruzza, A.; Scalisi, T.G.	Italy	Sapienza University of Rome/Niccolò Cusano University of Rome	Case study
[29]	Billeci, L.; Caterino, E.; Tonacci, A.; Gava, M.L.	Italy	National Research Council	Research
[30]	Narzisi, A.; Bondioli, M.; Pardossi, F.; Billeci, L.; Buzzi, M.C.; Buzzi, M.; Pinzino, M.; Senette, C.; Semucci, V.; Tonacci, A.; Uscidda, F.; Vagelli, B.; Giuca, M.R.; Pelagatti, S.	Italy	National Research Council/IRCCS Stella Maris/University of Pisa	Research
[31]	Troisi, J.; Autio, R.; Beopoulos, T.; Bravaccio, C.; Carraturo, F.; Corrivetti, G.; Cunningham, S.; Devane, S.; Fallin, D.; Fetissov, S.; Gea, M.; Giorgi, A.; Iris, F.; Joshi, L.; Kadzielski, S.; Kraneveld, A.; Kumar, H.; Ladd-Acosta, C.; Leader, G.; Mannion, A.; Maximin, E.; Mezzelani, A.; Milanesi, L.; Naudon, L.; Peralta Marzal, L.N.; Perez Pardo, P.; Prince, N.Z.; Rabot, S.; Roeselers, G.; Roos, C.; Roussin, L.; Scala, G.; Tuccinardi, F.P.; Fasano, A.	Italy; Finland; France; Ireland; USA; Netherlands;	University of Salerno/Tampere University/Bio-Modeling System/University of Naples Federico II/Promete srl/ASL Salerno/University Road/Massachusetts General Hospital/John Hopkins School of Public Health/University of Normandy/Medinok Spa/Utrecht University/Danone Nutricia Research/Université Paris-Saclay/National Research Council/Euformatics/EBRI, Salerno	Research
[32]	Magdalena, H.; Beata, K.; Justyna, P.; Agnieszka, K.-G.; Szczepara-Fabian, M.; Buczek, A.; Ewa, E.-W.	Poland	University of Silesia	Research
[33]	Pascucci, T.; Colamartino, M.; Fiori, E.; Sacco, R.; Coviello, A.; Ventura, R.; Puglisi-Allegra, S.; Turriziani, L.; Persico, A.M.	Italy	University of Messina/Sapienza University of Rome/IRCCS Fondazione Santa Lucia/IRCCS Neuromed	Research
[34]	Lombardo, S.D.; Battaglia, G.; Petralia, M.C.; Mangano, K.; Basile, M.S.; Bruno, V.; Fagone, P.; Bella, R.; Nicoletti, F.; Cavalli, E.	Italy	University of Catania/University Sapienza/IRCCS Neuromed	Research
[35]	Prosperi, M.; Guiducci, L.; Peroni, D.G.; Narducci, C.; Gaggini, M.; Calderoni, S.; Tancredi, R.; Morales, M.A.; Gastaldelli, A.; Muratori, F.; Santocchi, E.	Italy	IRCCS Stella Maris	Research
[36]	Lee, J.; Son, M.J.; Son, C.Y.; Jeong, G.H.; Lee, K.H.; Lee, K.S.; Ko, Y.; Kim, J.Y.; Lee, J.Y.; Radua, J.; Eisenhut, M.; Gressier, F.; Koyanagi, A.; Stubbs, B.; Solmi, M.; Rais, T.B.; Kronbichler, A.; Dragioti, E.; Vasconcelos, D.F.P.; Silva, F.R.P.; Tizaoui, K.; Brunoni, A.R.; Carvalho, A.F.; Cargnin, S.; Terrazzino, S.; Stickley, A.; Smith, L.; Thompson, T.; Shin, J.I.; Fusar-Poli, P.	Italy; Korea; USA; UK; Spain; Sweden; France; Austria; Brazil; Tunisia; Germany; Canada; Japan;	University Wonju/Yonsei University/Washington University/Gyeongsang National University/Yonsei University/Hankuk University/King’s College London/CIBERSAM/Karolinska Institute/IDIBAPS/Dunstable University/Bicêtre University Hospital/Universitat de Barcelona/ICREA/Instituto de Salud Carlos III/Maudsley NHS Foundation Trust/University of Padua/University of Toledo/University Innsbruck/Linköping University/Federal University of the Parnaiba Delta/Tunis El Manar University/University of São Paulo/University Hospital, LMU Munich/Centre for Addiction & Mental Health/University of Toronto/University of Piemonte Orientale/Södertörn University/National Institute of Mental Health, Tokyo/Anglia Ruskin University, Cambridge/University of Greenwich/OASIS Service, South London and Maudsley NHS Foundation Trust/University of Pavia	Research
[37]	Caria, A.; Ciringione, L.; de Falco, S.	Italy	University of Trento	Review
[38]	Fusar-Poli, L.; Cavone, V.; Tinacci, S.; Concas, I.; Petralia, A.; Signorelli, M.S.; Díaz-Caneja, C.M.; Aguglia, E.	Italy; Spain	University of Catania/Universidad Complutense	Review
[39]	Tanaka, S.; Komagome, A.; Iguchi-Sherry, A.; Nagasaka, A.; Yuhi, T.; Higashida, H.; Rooksby, M.; Kikuchi, M.; Arai, O.; Minami, K.; Tsuji, T.; Tsuji, C.	Japan; UK	Kanazawa University/Tokyo University/Kinjo University/University of Glasgow/University of Fukui	Research
[40]	Muratori, F.; Billeci, L.; Calderoni, S.; Boncoddo, M.; Lattarulo, C.; Costanzo, V.; Turi, M.; Colombi, C.; Narzisi, A.	Italy	IRCCS Stella Maris	Brief report
[41]	Masi, G.; Scullin, S.; Narzisi, A.; Muratori, P.; Paciello, M.; Fabiani, D.; Lenzi, F.; Mucci, M.; D’Acunto, G.	Italy	IRCCS Stella Maris	Research
[42]	Gulisano, M.; Barone, R.; Alaimo, S.; Ferro, A.; Pulvirenti, A.; Cirnigliaro, L.; Di Silvestre, S.; Martellino, S.; Maugeri, N.; Milana, M.C.; Scerbo, M.; Rizzo, R.	Italy	University of Catania	Research
[43]	Griffiths, A.J.; Hanson, A.H.; Giannantonio, C.M.; Mathur, S.K.; Hyde, K.; Linstead, E.	Usa	Chapman University	Research
[44]	Damiani, S.; Leali, P.; Nosari, G.; Caviglia, M.; Puci, M.V.; Monti, M.C.; Brondino, N.; Politi, P.	Italy	University of Pavia	Research
[45]	Keller, R.; Chieregato, S.; Bari, S.; Castaldo, R.; Rutto, F.; Chiocchetti, A.; Dianzani, U.	Italy	Adult Autism Center, Mental Health Department, Health Unit ASL Città di Torino/University of Turin	Research
[46]	Runge, K.; Tebartz van Elst, L.; Maier, S.; Nickel, K.; Denzel, D.; Matysik, M.; Kuzior, H.; Robinson, T.; Blank, T.; Dersch, R.; Domschke, K.; Endres, D.	Germany	University of Freiburg	Research
[47]	Fusar-Poli, L.; Ciancio, A.; Gabbiadini, A.; Meo, V.; Patania, F.; Rodolico, A.; Saitta, G.; Vozza, L.; Petralia, A.; Signorelli, M.S.; Aguglia, E.	Italy	University of Catania	Research
[48]	Leonardi, E.; Cerasa, A.; Famà, F.I.; Carrozza, C.; Spadaro, L.; Scifo, R.; Baieli, S.; Marino, F.; Tartarisco, G.; Vagni, D.; Pioggia, G.; Ruta, L.	Italy	National Research Council	Research
[49]	Riva, V.; Marino, C.; Piazza, C.; Riboldi, E.M.; Mornati, G.; Molteni, M.; Cantiani, C.	Italy; Canada	Scientific Institute IRCCS E. Medea/University of Toronto	Research
[50]	Bianco, V.; Finisguerra, A.; Betti, S.; D’Argenio, G.; Urgesi, C.	Italy	University of Udine	Research
